# Survey of basic medical researchers on the awareness of animal experimental designs and reporting standards in China

**DOI:** 10.1371/journal.pone.0174530

**Published:** 2017-04-05

**Authors:** Bin Ma, Jia-ke Xu, Wen-jing Wu, Hong-yan Liu, Cheng-kun Kou, Na Liu, Lulu Zhao

**Affiliations:** 1 Evidence-Based Medicine Center of Lanzhou University, School of Basic Medical Sciences, Lanzhou University, Lanzhou, China; 2 Key Laboratory of Evidence Based Medicine and Knowledge Translation of Gansu Province, Lanzhou, China; 3 The First Clinical Medical College of Lanzhou University, Lanzhou, China; Van Andel Institute, UNITED STATES

## Abstract

**Objective:**

To investigate the awareness and use of the Systematic Review Center for Laboratory Animal Experimentation’s (SYRCLE) risk-of-bias tool, the Animal Research: Reporting of In Vivo Experiments (ARRIVE) reporting guidelines, and Gold Standard Publication Checklist (GSPC) in China in basic medical researchers of animal experimental studies.

**Methods:**

A national questionnaire-based survey targeting basic medical researchers was carried in China to investigate the basic information and awareness of SYRCLE’s risk of bias tool, ARRIVE guidelines, GSPC, and animal experimental bias risk control factors. The EpiData3.1 software was used for data entry, and Microsoft Excel 2013 was used for statistical analysis in this study. The number of cases (n) and percentage (%) of classified information were statistically described, and the comparison between groups (i.e., current students *vs*. research staff) was performed using chi-square test.

**Results:**

A total of 298 questionnaires were distributed, and 272 responses were received, which included 266 valid questionnaires (from 118 current students and 148 research staff). Among the 266 survey participants, only 15.8% was aware of the SYRCLE’s risk of bias tool, with significant difference between the two groups (P = 0.003), and the awareness rates of ARRIVE guidelines and GSPC were only 9.4% and 9.0%, respectively; 58.6% survey participants believed that the reports of animal experimental studies in Chinese literature were inadequate, with significant difference between the two groups (P = 0.004). In addition, only approximately 1/3 of the survey participants had read systematic reviews and meta-analysis reports of animal experimental studies; only 16/266 (6.0%) had carried out/participated in and 11/266 (4.1%) had published systematic reviews/meta-analysis of animal experimental studies.

**Conclusions:**

The awareness and use rates of SYRCLE’s risk-of-bias tool, the ARRIVE guidelines, and the GSPC were low among Chinese basic medical researchers. Therefore, specific measures are necessary to promote and popularize these standards and specifications and to introduce these standards into guidelines of Chinese domestic journals as soon as possible to raise awareness and increase use rates of researchers and journal editors, thereby improving the quality of animal experimental methods and reports.

## Introduction

As an important bridge between basic medical research and clinical trials, animal experimental studies are important to validate the safety and efficacy of the interventions and to determine whether new interventions can be applied to clinical trials [1, 2]. A large number of published animal studies have varying degrees of defects in their experimental designs, implementation, and reporting methods, which seriously affects the authenticity and reliability of these animal studies, and poses difficulties in replicating results [3–7].

To address this problem, Animal Research: Reporting In Vivo Experiment (ARRIVE) guidelines [8] and the Gold Standard Publication Checklist (GSPC) [9] were published to standardize animal experimental reports, to ensure sufficient evaluation and utilization of animal experimental data, and to facilitate integrity and transparency during the review processes of basic medical research [10, 11]. Following the publication of these standards, to design more rigorous animal experimental designs and to effectively control risk of bias, Hooijmans and other researchers from the Systematic Review Centre for Laboratory Animal Experimentation (SYRCLE) in the Netherlands used Cochrane’s risk-of-bias tool (as recommended by the *Cochrane Handbook* [12]) to study, draft, and develop SYRCLE’s risk-of-bias tool for animal experimental studies, which was published in 2014 [13]. These guidelines have important implications for the scientific design, rigorous implementation, and regulatory reporting of animal experimental studies. Although these guidelines were introduced in China several years ago [10, 11], a previous study [14] demonstrated that the editorial staff of Chinese domestic journals that publish animal experimental studies have a limited awareness of this information and low implementation rates of the ARRIVE guidelines and GSPC. In addition to the journal editors, basic medical researchers have significant influence on the degree of bias in the design and implementation of animal experimental studies and on the adequacy of the reported results. Although a study by Fang et al. [15] in China conducted a relevant survey with animal experimental researchers in Lanzhou City, Gansu Province, China, the survey was limited to the awareness of the ARRIVE guidelines and GSPC.

Therefore, this study conducted a questionnaire-based survey to expand the scope of survey respondents and contents to comprehensively investigate awareness of the design and implementation of animal experimental methods as well as reporting standards and their use. This study identified problems and promoted the popularity of these guidelines to basic medical researchers to improve the quality of animal experimental studies.

## Materials and methods

### 2.1. Survey respondents

Survey respondents include current students (i.e., master and doctoral students in the graduate school) and research staff members (e.g., university faculties, researchers, experimental technicians, and clinicians) in the field of basic medical research. Convenience sampling was used and respondents were from six major geographic areas of China, including 23 medical institutes and affiliated hospitals in Northwest China, Southwest China, South China, North China, East China, and Northeast China.(S1 File)

### 2.2. Survey methods

With reference to relevant literature and combined with the details of SYRCLE’s risk-of-bias tool, the ARRIVE guidelines, and the GSPC, we designed the questionnaires in the form of single-answer and multiple-choice questions. The questionnaires (S2 File) were distributed through e-mail, the postal service, and on-site delivery and were collected and gathered by specialized investigators(XU Jia-ke, WU Wen-jing, LIU Hong-yan, KOU Cheng-kun) to ensure authenticity and legitimacy of the survey results. The survey was conducted from June to August 2016.

### 2.3. Survey contents

The survey contents included (1) general information about survey respondents (e.g., academic degree and occupation), time had spent in basic medical teaching or research, whether had participated or directed any projects on animal experimental studies, number of articles related to animal experimental studies read per month, and means to access relevant research progress; (2) awareness of the items of SYRCLE’s risk-of-bias tool; (3) status of relevant published reports on animal experimental studies; (4) whether had written or published papers on animal experimental studies, awareness and source of the ARRIVE guidelines and GSPC; and (5) awareness of systematic review and meta-analysis of animal experimental studies.

### 2.4. Ethical review

The Ethical Committee of School of Basic Medical Sciences, Lanzhou University, Lanzhou, Gansu, China, approved this survey. Given that this was a survey of opinion, with no risk of harm to respondents, we obtained informed consent verbally.

### 2.5. Statistical analysis

For this study, EpiData 3.1 software was used for data entry, and Microsoft Excel 2013 software was used for statistical analysis. The count data were described statistically using the number of cases (*n*) and percentage (%). Comparison of classified information (nongrade data) between current students and research staff was performed using chi-square test. The test level α was set to 0.05, and *P* < 0.05 was considered to be a statistically significant difference.

## Results

### 3.1. General information about survey respondents (see Table 1)

**Table 1:** General information about survey respondents.

Survey contents	Total *N* = 266	Current students *n* = 118 (44.4%)	Research staff *n* = 148 (55.6%)
**Time had spent in basic medical teaching or research**			
<5 year	152(57.1)	108(91.5)	44(29.7)
5–10 year	67(25.2)	9(7.6)	58(39.2)
10–20 year	41(15.4)	1(0.8)	40(27.0)
≥20 year	6(2.3)	0(0.0)	6(4.1)
**Participated or developed any projects on animal experimental studies**	239(89.8)	103(87.3)	136(91.9)
**Directed or Participated projects on animal experimental studies as the main researchers**	198(74.4)	75(63.6)	123(83.1)
**Number of articles related to animal experimental studies read per month**			
>20	12(4.5)	3(2.5)	9(6.1)
20–10	32(12.0)	17(14.4)	15(10.1)
10–5	95(35.7)	47(39.8)	48(32.4)
<5	127(47.7)	51(43.2)	76(51.4)
**Means to access relevant research progress**			
Medical databases	240(90.2)	107(90.7)	133(89.9)
Specialty journals	25(9.4)	12(10.2)	13(8.8)
Academic conferences	64(24.1)	28(23.7)	36(24.3)
Online databases	127(47.7)	58(49.2)	69(46.6)
other	5(1.9)	1(0.8)	4(2.7)

Of the 298 questionnaires distributed, 272 responses were received (91.3% recovery rate), which included 266 valid questionnaires (97.8% efficiency).

Among the 266 valid survey respondents, most respondents were students (42.5%, 113/266) and teachers or researchers (36.1%, 96/266), followed by experimental technicians (12.4%, 33/266) and clinicians (7.5%, 20/266). Most of the survey respondents held master’s degrees (55.6%, 148/266) or doctoral degrees (25.2%, 67/266).

More than half of the 266 survey respondents (57.1%, 152/266) spent less than five years in basic medical teaching or research; most of the survey respondents (89.8%, 239/266) developed or had participated in animal experimental studies; 74.4% (198/266) of the survey respondents were the main researchers who had participated in animal experimental studies.

Nearly half of the 266 survey respondents (47.7%, 127/266) read less than five articles pertaining to animal experimental studies per month. The vast majority of respondents accessed relevant research updates from medical databases (90.2%, 240/266), followed by respondents who accessed online databases (47.7%, 127/266) or attended academic conferences (24.1%, 64/266).

### 3.2. Awareness and knowledge of SYRCLE’s risk-of-bias tool and bias risk control factors (see Table 2)

**Table 2:** Awareness and knowledge of SYRCLE’s risk-of-bias tool and bias risk control factors.

Survey contents	Total *N* = 266	Current students *n* = 118 (44.4%)	Research staff *n* = 148 (55.6%)	*P* value
**Awareness and Knowledge of SYRCLE’s Risk-Of-Bias Tool**				
Has heard of it but does not understand detail and content	42(15.8)	10(8.5)	32(21.6)	0.003
Has heard of it and also understands detail and content	8(3.0)	3(2.5)	5(3.4)	0.972
**Considered the items of SYRCLE’s Risk-Of-Bias Tool was "very necessary"**				
1. Sequence generation	228(85.7)	103(87.3)	125(84.5)	0.314
2. Baseline characteristics	210(78.9)	98(83.1)	112(75.7)	0.143
3. Allocation concealment	107(40.2)	48(40.7)	59(39.9)	0.893
4. Random housing	82(30.8)	41(34.7)	41(27.7)	0.217
5. Blinding (caregivers and researchers)	110(41.4)	56(47.5)	54(36.5)	0.071
6. Random outcome assessment	172(64.7)	77(65.3)	95(64.2)	0.857
7.Blinding (outcome assessors)	158(59.4)	78(66.1)	80(54.1)	0.047
8.Incomplete outcome data	217(81.6)	98(83.1)	119(80.4)	0.580
9.Selective outcome reporting	132(49.6)	60(50.8)	72(48.6)	0.722

Of the 266 survey respondents, only 15.8% (42/266) had heard of SYRCLE’s risk-of-bias tool, with a statistically significant difference between groups (*P* = 0.003). Only 3.0% (8/287) understood and were familiar with SYRCLE’s risk-of-bias tool (without significant difference between groups, *P* = 0.972).

Among the items included in SYRCLE’s risk-of-bias tool, most survey respondents considered the first item (No. 1) (85.7%, 228/266) and the eighth item (No. 8) (81.6%, 217/266) to be very necessary. More than half of the survey respondents considered the second item (No. 2) (78.9%, 210/266), the seventh item (No. 7) (59.4%, 158/266), and the sixth item (No. 6) (64.7%, 172/266) to be very necessary. Less than half of the survey respondents considered that the third item (No. 3) (40.2%, 107), the fourth item (No. 4) (30.8%, 82/266), the fifth item (No. 5) (41.4%, 110/266), and the ninth item (No. 9) (49.6%, 132), to be very necessary. Except for current students (66.1%, 78/266) and research staff (54.1%, 80/266) who considered the sixth item (No. 6) to be very necessary (with significant difference, *P* = 0.047), the remaining items included in SYRCLE’s risk-of-bias tool held no significant difference between groups.

### 3.3. Investigation of existing literature in animal experiments (see Table 3)

**Table 3:** Investigation of existing literature in animal experiments.

Survey contents	Total *N* = 266	Current students *n* = 118 (44.4%)	Research staff *n* = 148 (55.6%)	*P* value
**Whether the reporting quality of the published animal experimental were adequate**				
adequate	156(58.6)	57(48.3)	99(66.9)	0.004
inadequate	110(41.4)	60(50.8)	50(33.8)	
**Reporting quality of Published animal experiments (the percentage of inadequate)**				
1 Background section				
1.1 Explain how and why the animal species and model being used can address the scientific objectives and, where appropriate, the study’s relevance to human biology	106(67.9)	36(63.2)	70(70.7)	0.005
1.2 Include sufficient scientific background (including relevant references to previous work) to understand the motivation and context for the study, and explain the experimental approach and rationale.	51(32.7)	23(40.4)	28(28.3)	0.906
2 Method section				
2.1 Experimental design-related information, including: The number of experimental and control groups. Any steps taken to minimise the effects of subjective bias when allocating animals to treatment (e.g.,randomisation procedure) and when assessing results (e.g., if done, escribe who was blinded and when). The experimental unit (e.g. a single animal, group, or cage of animals). A time-line diagram or flow chart can be useful to illustrate how complex study designs were carried out.	108(69.2)	36(63.2)	72(72.7)	0.003
2.2 Experimental procedure-related information, including: How (e.g., drug formulation and dose, site and route of administration, anaesthesia and analgesia used [including monitoring], surgical procedure, method of euthanasia). Provide details of any specialist equipment used, including supplier(s). When (e.g., time of day). Where (e.g., home cage, laboratory, water maze). Why (e.g., rationale for choice of specific anaesthetic, route of administration, drug dose used).	103(66.0)	38(66.7)	65(65.7)	0.051
2.3 Animal facilities and feeding and housing conditions, including: Housing (e.g., type of facility, e.g., specific pathogen free (SPF); type of cage or housing; bedding material; number of cage companions; tank shape and material etc. for fish). Husbandry conditions (e.g., breeding programme, light/dark cycle, temperature, quality of water etc. for fish, type of food, access to food and water, environmental enrichment). Welfare-related assessments and interventions that were carried out before, during, or after the experiment.	99(63.5)	30(52.6)	69(69.7)	0.000
2.4 Experimental animal-related information, including: Provide details of the animals used, including species, strain, sex, developmental stage (e.g., mean or median age plus age range), and weight (e.g., mean or median weight plus weight range). Provide further relevant information such as the source of animals, international strain nomenclature, genetic modification status (e.g. knock-out or transgenic), genotype, health/immune status, etc.	90(57.7)	25(43.9)	65(65.7)	0.000
2.5 Sample-size related information, including: Specify the total number of animals used in each experiment and the number of animals in each experimental group. Explain how the number of animals was decided. Provide details of any sample size calculation used. Indicate the number of independent replications of each experiment, if relevant.	76(48.7)	28(49.1)	48(48.5)	0.119
2.6 Statistical analysis-related information, including: Provide details of the statistical methods used for each analysis. Specify the unit of analysis for each dataset (e.g. single animal, group of animals, single neuron). Describe any methods used to assess whether the data met the assumptions of the statistical approach.	70(44.9)	16(28.1)	54(54.5)	0.000
2.7 Experimental outcomes-related information, including: define the primary and secondary experimental outcomes assessed (e.g., cell death, molecular markers, behavioural changes).	32(20.5)	3(5.3)	29(29.3)	0.000
3 Results section				
3.1Adverse reaction-related information, including: Give details of all important adverse events in each experimental group. Describe any modifications to the experimental protocols made to reduce adverse events.	85(54.5)	30(52.6)	55(55.6)	0.041
3.2Data analysis-related information, including: Report the number of animals in each group included in each analysis. Report absolute numbers (e.g.10/20, not 50%). If any animals or data were not included in the analysis, explain why.	79(50.6)	18(31.6)	61(61.6)	0.000
3.3Baseline data-related information, including: a. Relevant characteristics and health status in each experimental group animals (e.g., weight, and microbiological status etc.) before treatment or testing (this information can often be tabulated).	53(34.0)	15(26.3)	38(38.4)	0.009
3.4Outcomes and estimation-related information, including: a. the results for each analysis carried out, with a measure of precision (e.g., standard error or confidence interval).	37(23.7)	16(28.1)	21(21.2)	0.883
4 Discussion section				
4.1 Limitation of the study, including: any potential sources of bias, any limitations of the animal model, and the imprecision associated with the results	76(48.7)	23(40.4)	53(53.5)	0.003
4.2 Generalisability/translation-related information, including: whether, and how, the findings of this study are likely to translate to other species or systems, including any relevance to human biology.	57(36.5)	16(28.1)	41(41.4)	0.005
4.3Taking account of the student objectives, hypotheses, and the current theory of other literature and relevant studies when interpreting the study results	47(30.1)	16(28.1)	31(31.3)	0.117
4.4 Describe any implications of your experimental methods or findings for the replacement, refinement, or reduction (the 3Rs) of the use of animals in research	44(28.2)	12(21.1)	32(32.3)	0.013
4.5 Funding-related information, including: list all funding sources (including grant number) and the role of the funder(s) in the study.	7(4.5)	1(1.8)	6(6.1)	0.105

Of the 266 survey respondents, 58.6% (156/266) considered existing literature on animal experimental studies to be inadequate (significant difference between groups, *P* = 0.004).

More than 50% of the survey respondents believed that existing literature on animal experimental studies was inadequate in the following aspects:

- Background section: “Explain how and why the animal species and model being used can address the scientific objectives and, where appropriate, the study’s relevance to human biology” (67.9%, 106/266; significant difference between groups, *P* = 0.005).
- Method section: (a) “Experimental design-related information” (69.2%, 108/266; significant difference between groups, *P* = 0.003), (b) “Experimental procedure-related information” (66.0%, 103/266; no significant difference between groups, *P* = 0.051), (c) “Animal facilities and feeding and housing conditions” (63.5%, 99/266; significant difference between groups, *P* = 0.000), and (d) “Experimental animal-related information” (57.7%, 90/266; significant difference between groups, *P* = 0.000).
- Results section: (a) “Adverse reaction-related information” (54.5%, 85/266; significant difference between groups, *P* = 0.041) and (b) “Data analysis-related information” (50.6%, 79/266; significant difference between groups, *P* = 0.000).

Lower than 50% and More than 30% of the survey respondents considered existing literature on animal experimental studies to be inadequate in the following aspects:

- Background section: “Interpretation of study objective(s), contents, experimental methods, and basic principles” (32.7%, 51/266; no significant difference between groups, *P* = 0.906).
- Method section: (a) “Sample-size related information” (48.7%, 76/266; no significant difference between groups, *P* = 0.119) and (b) “Statistical analysis-related information” (44.9%, 70/266; significant difference between group, *P* = 0.000).
- Results section: “Baseline data-related information including Relevant characteristics and health status in each experimental group animals before treatment or testing” (34.0%, 53/266; significant difference between groups, *P* = 0.009).
- Discussion section: (a) “Limitations of the study” (48.7%, 76/266; significant difference between group, *P* = 0.003), (b) “Generalisability/translation-related information” (36.5%, 57/266; significant difference between group, *P* = 0.005), and (c) “Taking account of the student objectives, hypotheses, and the current theory of other literature and relevant studies when interpreting the study results” (30.1%, 47/266; no significant difference between groups, *P* = 0.117).

### 3.4. Awareness of the ARRIVE guidelines and GSPC(see Table 4)

**Table 4:** Survey of the awareness rates of the ARRIVE guidelines and GSPC.

Survey contents	Total *N* = 266	Current students *n* = 118 (44.4%)	Research staff *n* = 148 (55.6%)	*P* value
**Relevant writing and publication of animal experimental studies**				
Participated in writing manuscripts of animal experimental studies	143 (53.8)	45 (38.1)	98 (66.2)	0.000
Published animal experimental studies	120 (45.1)	28 (23.7)	92 (62.2)	0.000
**Wrote and reported animal experimental results mainly based on**				
1. References of relevant published studies	218 (82.0)	95 (80.5)	123 (83.1)	0.584
2. Requirements of journal editors	97 (36.5)	48 (40.7)	49 (33.1)	0.203
3. The instruction to the authors provided by targeted journals	86 (32.3)	43 (36.4)	43 (29.1)	0.201
4. Personal preferences and choices	52 (19.5)	23 (19.5)	29 (19.6)	0.983
5. Others	10 (3.8)	6 (5.1)	4 (2.7)	0.310
**Awareness of the ARRIVE guidelines and GSPC**				
1. Heard of the ARRIVE guidelines	25 (9.4)	7 (5.9)	18 (12.2)	0.084
2. Understood and were familiar with the ARRIVE guidelines	8 (3.0)	3 (2.5)	5 (3.4)	0.692
3. Heard of GSPC	24 (9.0)	6 (5.1)	18 (12.2)	0.045
4. Understood and were familiar with GSPC	9 (3.4)	5 (4.2)	4 (2.7)	0.492
5. Believed it was necessary to develop a checklist to report results of animal experimental studies and to standardize the report of findings	219 (82.3)	96 (81.4)	123 (83.1)	0.710

Among the 266 survey respondents, 53.8% (143/266) and 45.1% (120/266) of the survey respondents participated in animal experimental studies and published animal experimental papers, respectively (significant difference between groups, *P* < 0.05). When preparing and reporting animal experimental results, the majority of survey respondents (82.0%, 218/266) primarily referred to publications of similar studies, followed by reference to the editorial requirements of the target journals (36.5%, 97/266) and the target journal’s guidelines (given in the introduction to authors, 32.3%, 86/266).

Of the 266 survey respondents, few respondents had heard of the ARRIVE guidelines (9.4%, 25/266) and GSPC (9.0%, 24/266), and very few respondents understood or were familiar with the ARRIVE guidelines (3.0%, 8/266) and GSPC (3.4%, 9/266). Most of the survey respondents (82.3%, 219/266), however, considered the necessity of studying animal experimental checklists to standardize the reporting of results and manuscript preparation.

### 3.5. Awareness of systematic reviews and meta-analysis of animal experimental studies (see Table 5)

**Table 5:** Awareness of systematic review and meta-analysis of animal experimental studies.

Survey contents	Total *N* = 266	Current students *n* = 118 (44.4%)	Research staff *n* = 148 (55.6%)	*P* value
1. Have you heard of systematic review/ meta-analysis?	206 (77.4)	89 (75.4)	117 (79.1)	0.482
2. Have you read any systematic review/ meta-analysis of animal experiments?	83 (31.2)	38 (32.2)	45 (30.4)	0.753
3. Have you conducted or participated in systematic review/meta-analysis of animal experiments?	16 (6.0)	9 (7.6)	7 (4.7)	0.323
4. Have you published systematic review/ meta-analysis of animal experiments?	11 (4.1)	4 (3.4)	7 (4.7)	0.586
5.Have you considered systematic review/meta-analysis to be an effective way to improve the value of animal experiments to guide clinical research?	187 (70.3)	86 (72.9)	101 (68.2)	0.411

Although most of the respondents (77.4%, 206/266) had heard of systematic reviews and meta-analysis methods, of the 266 survey respondents, only about a third (31.2%, 83/266) had read about these published systematic reviews or meta-analysis of animal experimental studies (see Table 2). Very few respondents had conducted (6.0%, 16/266) or published (4.1%, 11/266) systematic reviews or meta-analysis of animal experimental studies. Most of the survey respondents, however, believed that systematic review or meta-analysis would be an effective way to increase the value of animal experimental studies to clinical research.

## Discussion

Animal experimental studies not only provide preliminary validation of the safety and efficacy of interventions but also provide scientific evidence to determine whether new interventions can enter the clinical research phase. This validation must be based on scientific design, rigorous implementation, and reports with standardized results.

This study’s survey of animal experimental methods showed that most survey respondents believed that “randomization (85.7%),” “baseline characteristics (78.9%),” and “incomplete data reporting (81.6%)” play a very important role in the design of animal experimental studies. Less than 50% of the survey respondents, however, believed that the implementation of “allocation concealment,” “animal randomization,” and “blindness” (e.g., focusing on the animal breeders, researchers, and personnel performed outcome measurement) or measures to avoid “selective outcome reporting” were very important in animal experimental studies. Allocation concealment hides the generated random sequence number to ensure that participants do not know the random sequence number. This measure is used to control and reduce selection bias along with animal randomization [16]. Some international studies have shown that implementing strict randomization to generate random sequence numbers but not applying allocation concealment results in an exaggeration of treatment efficacy by 30–41% [16]. Similar to clinical studies, implementation of blindness in animal experimental studies is essential, especially for the subjective outcome measurements of some indicators to effectively avoid and reduce the bias in implementation or measurement [13, 17]. In contrast to clinical studies, “animal randomization” and “evaluation of random outcomes” are very important to control for risk of laboratory bias in animal experimental studies. Results of this study, however, showed that only 30% of the survey respondents believed “animal randomization” to be very important, and only 50% of the survey respondents considered the “evaluation of random outcomes” to be very important. Regarding different housing conditions of the experimental animals, different light intensity and temperatures of the animal facilities have important impacts on the results of these animal studies. For example, numerous studies have shown that animal cages placed at the higher position of the rack are exposed to four times the light intensity compared with the animal cages placed at the bottom of the rack [13,18]. Slight alterations of light intensity affect and change the reproduction and behavior of the animals [13, 19–21]. In addition, temperature variations inside animal cages result from their placement at different levels of the rack (cages at the top of the rack are typically 5°C higher than cages at the bottom of the rack) [13, 18, 22, 23]. Conversely, if animals are not randomized, researchers may anticipate the behaviors of each group of animals, resulting in implementation bias. Moreover, in animal experimental studies, random selection of experimental animals is very important for the evaluation and measurement of study outcomes. Because most living beings have circadian rhythms, such as lipid (compound) metabolism, neurotransmitter levels, and pharmacokinetics, which affect the variations of cycle and circadian rhythms of animals [13, 24–26], measurement bias may be introduced when sample evaluations and measurements are conducted only in certain periods of time without applying randomization during the measurement.

SYRCLE’s risk-of-bias tool not only helps researchers to standardize the design of animal experimental studies but also provides a specific set of tools to assess authenticity in these experiments. In this study, the survey of experimental methods used in animal studies was based on this assessment tool. In 2014, our research team had introduced and described SYRCLE’s risk-of-bias tool for animal experimental studies in detail [11], but the results of this current survey show that only 15.8% of the survey respondents had heard of SYRCLE, and only 3% of the survey respondents understood and were familiar with this assessment tool. This low level of awareness might explain the overall low quality of animal experimental studies in China. On the basis of these findings, it was necessary to further increase the promotion of SYRCLE’s risk-of-bias tool in China to encourage basic medical researchers to design animal experimental studies more scientifically and rigorously to improve the authenticity of these experiments.

In this study, a survey of reporting standards of animal experimental studies showed that less than 10% of the survey respondents had heard of the ARRIVE guidelines and GSPC, and less than 5% of the survey respondents understood and were familiar with these two reporting standards. When writing manuscripts detailing animal experimental design, most of the respondents referred primarily to published literature of relevant studies (82.0%, 218/266), the requirements of the target journals (36.5%, 97/266), and the introduction to authors provided by the target journal’s guidelines (32.3%, 86/266). A previous study [14], however, has shown that the 240 domestic journals in China that have published animal experimental studies do not mention the ARRIVE guidelines or GSPC in their journal guidelines. In addition, the editorial staff of the relevant journals had very low awareness of these reporting standards. This lack of awareness may explain the inadequacy of reporting animal experimental results in the majority of studies conducted in China, which was confirmed in this study. When the 266 survey respondents read the animal experimental literature, more than 50% believed that the existing literature on animal experimental studies was inadequate, primarily in the methods and results sections. Since these reporting standards were issued, the ARRIVE guidelines and GSPC have been included in the introduction to authors provided by 317 international journals [27] and were endorsed and recommended by International Committee of Medical Journal Editors and EQUATE Network [28]. A study [29] showed that the use of ARRIVE guidelines and GSPC can improve the quality of reports on animal experimental studies. The ARRIVE guidelines and GSPC provide important guidance about manuscript preparation for animal experimental studies, which will enable readers to accurately and clearly understand the content of these manuscripts. Moreover, these guidelines provide important information required for the replication of the animal experiments [8] to ensure that the information about these animal experimental studies will be assessed adequately and utilized to facilitate integrity and transparency of the basic medical research review process and to avoid wasting medical resources [30]. Therefore, we highly recommend introducing the ARRIVE guidelines and GSPC to Chinese domestic journals that publish animal experimental studies and related studies to improve the awareness and actual use rate of these reporting standards.

A limitation of our study was used convenience sampling rather than random sampling, even though convenience sampling has a high rate of feedback. In addition, participants in our survey study were from six major geographic areas of China, but the number of participants relative to the estimated size of the target population did not evaluated which may affected the outcome of our survey study.

## Conclusions

Basic medical researchers in China had low awareness and use of SYRCLE’s risk-of-bias tool, the ARRIVE guidelines, and GSPC in animal experimental studies conducted in China. Therefore, we proposed taking specific measures to promote and popularize these guidelines and to introduce these guidelines in Chinese domestic journals as soon as possible. In so doing, we hope to improve awareness and actual use rates of these guidelines by basic medical researchers and journal editors, thereby improving the quality of animal experimental methods and reporting standards.

## Supporting information

S1 File Name of medical institutes and affiliated hospital in our survey study. (DOCX)

S2 File The questionnaire used in our survey study. (DOCX)
