# Supplementary material for: Survey of basic medical researchers on the awareness of animal experimental designs and reporting standards in China
Source: PLoS One. 2017 Apr 5;12(4):e0174530. doi: 10.1371/journal.pone.0174530 (PMC5381903; doi:10.1371/journal.pone.0174530)
Supplement: S1 File — (DOCX) [file pone.0174530.s001.docx]

**S1 file Name of medical institutes and affiliated hospital in our survey study**

| **Geographic areas of China** | **Name of medical institutes and affiliated hospital** |
| --- | --- |
| Northwest China | Lanzhou University School of Medicine |
|  | Gansu University of Chinese Medicine |
|  | School of Medicine, Northwest University for Nationalities |
|  | Nixiang Medical University |
|  | Medical College of Qinhai University |
|  | Xi’an Jiaotong University health science center |
|  | Xi’an Medical College |
| Southwest China | West China Hospital, Sichuan University |
|  | School of Preclinical and Forensic Medicine, Sichuan University |
|  | North Sichuan Medical College |
| South China | Zhongshan School of Medicine, Sun Yat-sen University |
|  | the First Affiliated Hospital, Sun Yat-sen University |
| North China | Peking University School of Basic Medical Sciences |
|  | Peking University Cancer Hospital |
|  | Chinese Academy of Medical Sciences ＆ Peking Union Medical College |
|  | Institute of Biophysics, Chinese Academy of Sciences |
|  | Xiyuan Hospital, China Academy of Chinese Medical Sciences |
|  | Tianjin Medical University |
|  | Pingjing Hospital |
|  | North China University of Science and Technology Affiliated Hospital |
|  | Hebei Medical University |
| Northeast China | Harbin Medical University |
|  | Norman Bethune Health Science Center of Jinlin University |
