# Supplementary material for: Survey of basic medical researchers on the awareness of animal experimental designs and reporting standards in China
Source: PLoS One. 2017 Apr 5;12(4):e0174530. doi: 10.1371/journal.pone.0174530 (PMC5381903; doi:10.1371/journal.pone.0174530)
Supplement: S2 File — (DOCX) [file pone.0174530.s002.docx]

**动物实验相关报告标准的认知情况调查**

尊敬的老师：

您好！非常感谢您抽出宝贵的时间填写这份问卷！本调查的目的是为了了解基础医学研究人员对“动物实验报告标准” 的认知度和需求情况。请在您认可的选项前的字母上打**√**或在横线处填写，选择题如无特殊说明均为单选题。您的回答将是对我们课题最大的支持与帮助！谢谢！

本课题为国家自然科学基金青年项目的资助（编号：81303147）和兰州大学中央高校基本业务费资助（编号：lzujbky-2016-67）。

兰州大学循证医学中心**·**动物实验报告标准建立课题组

二零一六年五月

**基本信息**

年龄 ；专业 ；职称 ；

最高学历（□A:博士；□B:硕士；□C:本科；□D：其他）；

职业（□A:教师/科研人员；□B:实验教学/技术人员；□C:临床医生；□D：在读硕士/博士研究生）；

单位 ；

1. 您从事基础医学教学/研究多长时间？

□A：5 年以下；□B：5-10 年； □C：10-20 年；□D：20 年以上

1. 您是否开展/参与过动物实验（包括活体动物实验或体外细胞学实验等）相关研究?（□A:是；□B:否）
2. 您是否以主要成员参加过动物实验（包括活体动物实验或体外细胞学研究等）相关研究课题？（□A:是；□B:否）
3. 您是否主持过动物实验（包括活体动物实验或体外细胞学研究等）相关研究课题？（□A:是；□B：否）
4. 您平均每月阅读基础医学研究或动物实验研究领域相关论文多少篇？

□A: 20篇以上；□B：20-10 篇； □C：10-5 篇；□D：5篇以下

1. 您通常通过何种途径获取您所在专业的最新或重要的研究进展（多选）

□A：医学数据库；□B：纸版专业期刊；□C：学术会议；

□D：网站搜索；□E：其他

1. 在动物实验（限定为体内实验）设计、实施和资料分析阶段，请您选择以下措施的必要性：

①使用随机分组的方法确定实验动物的入组

□非常必要； □不一定，依据情况而定；□没有必要

理由

②随机分组人员和实验实施人员不得为同一人

□非常必要； □不一定，依据情况而定；□没有必要

理由

③选择基线特征相近的动物入组，或对混杂因素进行调整

□非常必要； □不一定，依据情况而定；□没有必要

理由

④研究者在动物房中随机安置笼子或动物

□非常必要； □不一定，依据情况而定；□没有必要

理由

⑤采用一定的手段，使得动物饲养者和研究者不知晓动物接受何种干预措施

□非常必要； □不一定，依据情况而定；□没有必要

理由

⑥采用一定的手段，使得结果评价者不知晓动物接受何种干预措施

□非常必要；□不一定，依据情况而定；□没有必要

理由

⑦随机选择纳入实验的动物用于研究结果的评估和测量

□非常必要； □不一定，依据情况而定；□没有必要

理由

⑧详细记录并报告在每个干预组下（与最初分组的总数相比）失访、排除以及任何重新纳入分析的数据及其原因

□非常必要； □不一定，依据情况而定；□没有必要

理由

⑨严格遵循研究计划书说明，报告所有预定的结局指标

□非常必要； □不一定，依据情况而定；□没有必要

理由

1. 您是否参与撰写动物实验相关的论文（□A:是；□B：否）
2. 您是否发表过动物实验相关的论文（□A:是；□B：否）
3. 您在撰写或报告一项动物实验研究结果相关内容的主要依据是（可多选）：

□A:根据投稿杂志的稿约；□B:根据期刊编辑的要求；

□C:根据自己的喜好和选择；□D:参考已发表的同类研究

□E:其他：

1. 您在阅读已发表的动物实验相关文献时，其报告的内容是否足以让您对该研究的实施全过程及其存在的问题得到充分了解？（□A:是；□B:否）

如您第11题选择的是B，请继续回答第12题。反之，直接跳转到第13题。

1. 您觉得当前已发表的动物实验相关文献，在“背景、方法、结果和讨论”部分报告不充分主要体现在哪些方面？（可多选）

- **背景部分**：

□A：当前研究的现状（既往工作的相关参考文献）；

□B：研究目的和内容，实验方法和基本原理的解释；

□C：所用动物种类和模型的选择依据，该研究与人体生物学的关联程度；

- **方法部分：**

□A：实验设计相关信息，包括□实验组和对照组的数量；□具体随机方法，是否施盲及施盲对象等偏倚风险控制措施；□实验单位；□时线图或流程图解释复杂的研究设计的实施过程；

□B：实验步骤相关信息，包括□干预措施信息（如药物配方和剂量，给药部位和途径，麻醉镇痛药物的应用和监测，手术步骤，动物处死方法等），提供所使用的任何专业设备的详细信息，包括供应商；□实验实施日期；□实施场所如饲养笼、实验室和水迷宫等；

□C：实验动物相关信息，包括□研究动物的详细资料，包括种类、品系、雌雄、发育阶段（年龄均值或中位数）和体重（均值或中位数及其范围）；□进一步的相关信息，如动物来源、国际命名、遗传修饰状态（如基因敲除或转基因）、基因型、健康／免疫状况、未使用药物或未进行测试和先前的程序等；

□D：饲养场所和饲养信息，包括□饲养场所（如设备类型、无特定病原、舍类型、垫底材料、同笼同伴数量等）；□饲养条件（如繁殖计划、光／暗周期、温度和水质等）；□实验前、中和后期动物福利有关的评估和干预

□E：样本量相关信息，包括□所需样本量的算法及计算公式；□实验中使用的动物总数和每个实验组中分配的动物数；

□F：实验结果相关信息，界定主要和次要实验测量指标的评估（如细胞死亡、分子标记和行为改变）；

□G：统计方法相关信息，包括□每种分析所使用统计方法的详细信息；□每个数据集的分析单位（如单个动物、一组动物和单神经元）；□描述用来评估数据是否满足统计学方法的假设及所采用的任何方法

- **结果部分：**

□A：报告每个实验组治疗或测试前动物的有关特征和健康状况（如体重、微生物状况和药物测试），以表格形式表示；

□B：数字分析相关信息，包括□报告进入每一项分析中每组的动物数量，报告绝对数；□对于分析中未纳入的任何动物或数据，需说明原因；

□C：报告每一项分析的结果及精确度测量（如标准误或置信区间）；

□D：不良反应相关信息，包括□每个实验组所有重要不良反应详细信息；□描述为减少不良反应而对实验计划书所做出的修改；

- **讨论部分：**

□A：解释结果时考虑研究目的、假设以及文献报道的当前的理论和其他相关的研究

□B：评价研究的局限性，包括造成偏倚的任何潜在来源，动物模型的局限性以及与结果相关的不精确性；

□C：描述该研究方法或研究发现对于替代、优化或减少动物使用（３Ｒ原则）的意义

□D：评论是否或如何使本研究成果转化到其他物种或系统，包括与人体生物学相关的研究

□E：列出本研究涉及的所有资金来源（包括授权号）和研究资助者及其作用

1. 您觉得是否有必要研发动物实验研究报告的清单，以统一规范其论文的报告/撰写（□A:是；□B:否）
2. 您是否了解和熟悉ARRIVE清单（A:是；B：否）
3. 您是否听说过GSPC(Gold Standard Publication Checklist)清单

A:是 (途径：□学术讲座；□同行介绍；□文献阅读；□投稿时的期刊稿约；□相关培训班；□其他 )

B:否

1. 您是否了解和熟悉GSPC清单（□A:是；□B:否）
2. 您是否听说过系统评价/Meta-分析这种研究方法

A:是 (途径：□学术讲座；□同行介绍；□文献阅读；□其他 )

B:否

1. 您是否阅读过动物实验系统评价/Meta-分析研究（□A:是；□B:否）
2. 您是否开展/参与过动物实验系统评价/Meta-分析研究（□A:是；□B:否）
3. 您是否发表过动物实验系统评价/Meta-分析研究（□A:是；□B:否）
4. 您是否认为系统评价/Meta-分析是提升动物实验对临床研究指导价值的有效途径（□A:是；□B:否）

**Survey of basic medical researchers on the awareness of animal experimental designs and reporting standards in China**

Dear all:

Thank you very much for taking the time to fill in this questionnaire! The purpose of this study was to understand the awareness and needs of basic medical researchers for the "animal reporting standards". Your answer will be the greatest support and help to our project! Thank you.

The survey was supported by National Natural Science Foundation of China (Grant No. 81303147 ) and the Fundamental Research Funds for the Central Universities(Number: lzujbky-2016-67).

Evidence-based Medicine Center of Lanzhou University

2016.5

**Basic information**

Age ；Major ；Title ；

Degrees（□A: doctoral degrees；□B: master’s degrees；□C:本科；□D：其他）；

Occupation（□A: teachers/researchers；□B: experimental technicians；□C: clinicians；□D:students）；

Unit ；

1. Time had spent in basic medical teaching or research

□A：＜5 y；□B：5y-10 y； □C：10y-20y；□D：＞20 y

1. Participated or developed any projects on animal experimental studies（□A:yes；□B:no）
2. Directed or Participated projects on animal experimental studies as the main researchers（□A:yes；□B:no）

Number of articles related to animal experimental studies read per month

□A: ＞20；□B：20-10； □C：10-5；□D：＜5

1. Means to access relevant research progress（Multiselect）

□A：Medical databases；□B：Specialty journals；□C：Academic conferences；

□D：Online databases；□E：other

1. In the design, implementation and data analysis phase of animal experiments (defined as in vivo experiments), please select the following measures：

① Sequence generation

□very necessary； □Not necessarily, depending on the circumstances；□unecessary

② Allocation concealment

□very necessary； □Not necessarily, depending on the circumstances；□unecessary

③Baseline characteristics

□very necessary； □Not necessarily, depending on the circumstances；□unecessary

④ Random housing

□very necessary； □Not necessarily, depending on the circumstances；□unecessary

⑤Blinding（caregivers and researchers）

□very necessary； □Not necessarily, depending on the circumstances；□unecessary

⑥Random outcome assessment

□very necessary； □Not necessarily, depending on the circumstances；□unecessary

⑦Blinding（outcome assessors）

□very necessary； □Not necessarily, depending on the circumstances；□unecessary

⑧Incomplete outcome data

□very necessary； □Not necessarily, depending on the circumstances；□unecessary

⑨Selective outcome reporting

□very necessary； □Not necessarily, depending on the circumstances；□unecessary

1. Awareness and Knowledge of SYRCLE’s Risk-Of-Bias Tool：

A□: Has heard of it but does not understand detail and content

B□: Has heard of it and also understands detail and content

1. Participated in writing manuscripts of animal experimental studies（□A:yes；□B:no）
2. Published animal experimental studies（□A:yes；□B:no）
3. Wrote and reported animal experimental results mainly based on（Multiselect）：

□A: References of relevant published studies；□B: Requirements of journal editors；□C: The instruction to the authors provided by targeted journals；□D: Personal preferences and choices ；□E:other

1. Whether the reporting quality of the published animal experimental were adequate？（□A:yes；□B:no）

If you choose B in the 10 question, please continue to answer the 11 question. Instead, jump directly to the 12 question。

1. What do you think is the most important aspect of the published literature on animal testing in the context, method, results and discussion section？（可多选）

- **Background section**：

□A：Explain how and why the animal species and model being used can address the scientific objectives and, where appropriate, the study’s relevance to human biology

□B：Include sufficient scientific background (including relevant references to previous work) to understand the motivation and context for the study, and explain the experimental approach and rationale.

1.2

- **Method section：**

□A：Experimental design-related information, including□The number of experimental and control groups；□Any steps taken to minimise the effects of subjective bias when allocating animals to treatment (e.g.,randomisation procedure) and when assessing results (e.g., if done, escribe who was blinded and when)；□The experimental unit (e.g. a single animal, group, or cage of animals)；□A time-line diagram or flow chart can be useful to illustrate how complex study designs were carried out；

□B：Experimental procedure-related information, including:□How (e.g., drug formulation and dose, site and route of administration, anaesthesia and analgesia used [including monitoring], surgical procedure, method of euthanasia). Provide details of any specialist equipment used, including supplier(s)；□When (e.g., time of day)；□Where (e.g., home cage, laboratory, water maze)；□Why (e.g., rationale for choice of specific anaesthetic, route of administration, drug dose used)

□C：Animal facilities and feeding and housing conditions, including:□Housing (e.g., type of facility, e.g., specific pathogen free (SPF); type of cage or housing; bedding material; number of cage companions; tank shape and material etc. for fish)；□Welfare-related assessments and interventions that were carried out before, during, or after the experiment.

□D：Experimental animal-related information, including:□Provide details of the animals used, including species, strain, sex, developmental stage (e.g., mean or median age plus age range), and weight (e.g., mean or median weight plus weight range)；□Provide further relevant information such as the source of animals, international strain nomenclature, genetic modification status (e.g. knock-out or transgenic), genotype, health/immune status, etc

□E：Sample-size related information, including:□Specify the total number of animals used in each experiment and the number of animals in each experimental group；□Explain how the number of animals was decided. Provide details of any sample size calculation used；

□F：Experimental outcomes-related information, including: define the primary and secondary experimental outcomes assessed (e.g., cell death, molecular markers, behavioural changes)；

□G：Statistical analysis-related information, including:□Provide details of the statistical methods used for each analysis；□Specify the unit of analysis for each dataset (e.g. single animal, group of animals, single neuron)；□Describe any methods used to assess whether the data met the assumptions of the statistical approach

**Results section：**

□A：Outcomes and estimation-related information, including:a. the results for each analysis carried out, with a measure of precision (e.g., standard error or confidence interval)；

□B：Data analysis-related information, including:□Report the number of animals in each group included in each analysis. Report absolute numbers (e.g.10/20, not 50%)；□If any animals or data were not included in the analysis, explain why；

□C：Baseline data-related information, including: Relevant characteristics and health status in each experimental group animals (e.g., weight, and microbiological status etc.) before treatment or testing (this information can often be tabulated).；

□D：Adverse reaction-related information, including:□Give details of all important adverse events in each experimental group；□Describe any modifications to the experimental protocols made to reduce adverse events.

- **Discussion section：**

□A：Limitation of the study, including: any potential sources of bias, any limitations of the animal model, and the imprecision associated with the results

□B：Generalisability/translation-related information, including: whether, and how, the findings of this study are likely to translate to other species or systems, including any relevance to human biology；

□C：Taking account of the student objectives, hypotheses, and the current theory of other literature and relevant studies when interpreting the study results

□D：Describe any implications of your experimental methods or findings for the replacement, refinement, or reduction (the 3Rs) of the use of animals in research

□E：Funding-related information, including: list all funding sources (including grant number) and the role of the funder(s) in the study.

12. Believed it was necessary to develop a checklist to report results of animal experimental studies and to standardize the report of findings（□A:yes；□B:no

13.Heard of the ARRIVE guidelines（□A:yes；□B:no）

14.Understood and were familiar with the ARRIVE guidelines（□A:yes；□B:no）

15.Heard of the GSPC（□A:yes；□B:no）

16.Understood and were familiar with the GSPC（□A:yes；□B:no）

17.Have you heard of systematic review/ meta-analysis（□A:yes；□B:no）

18.Have you read any systematic review/ meta-analysis of animal experiments?（□A:yes；□B:no）

19.Have you conducted or participated in systematic review/meta-analysis of animal experiments?（□A:yes；□B:no）

20.Have you published systematic review/ meta-analysis of animal experiments?（□A:yes；□B:no）

21.Have you considered systematic review/meta-analysis to be an effective way to improve the value of animal experiments to guide clinical research?（□A:yes；□B:no）
